# Iatrogenic metastasis of gastric adenocarcinoma to the abdomen

**DOI:** 10.11604/pamj.2023.44.81.38972

**Published:** 2023-02-09

**Authors:** Linna Lv, Zhuangli Tang

**Affiliations:** 1 Linping Campus, The Second Affiliated Hospital of Zhejiang University School of Medicine, Hangzhou 311100, China,; 2 The Second Affiliated Hospital of Zhejiang University School of Medicine, Hangzhou 310009, China

**Keywords:** Gastric adenocarcinoma, drain, iatrogenic metastasis

## Image in medicine

One senior man in his late 70s presented to our clinic in early November, 2022 with numerous erythematous nodules on the right upper abdomen for one month. Some three months ago, he was diagnosed with antrum adenocarcinoma and underwent radical gastrectomy. Unfortunately, he complained of abdominal distension within one-month posterior to the operation. Computed tomography of the abdomen indicated multiple peritoneal nodules accompanied by ascites and subsequent paracentesis was done. Shortly after the removal of the drainage, he stumbled across several subtle nodules where the drainage had been previously placed. By degrees the nodules enlarged and satellite lesions were noticed peripherally. Skin biopsy was arranged and the histopathologic findings were consistent with cutaneous metastasis of gastric adenocarcinoma. On the basis of the poor condition, the patient was prescribed with oral Tirelizumab. Upon the last visit this month, the nodules diminished significantly.

**Figure 1:**
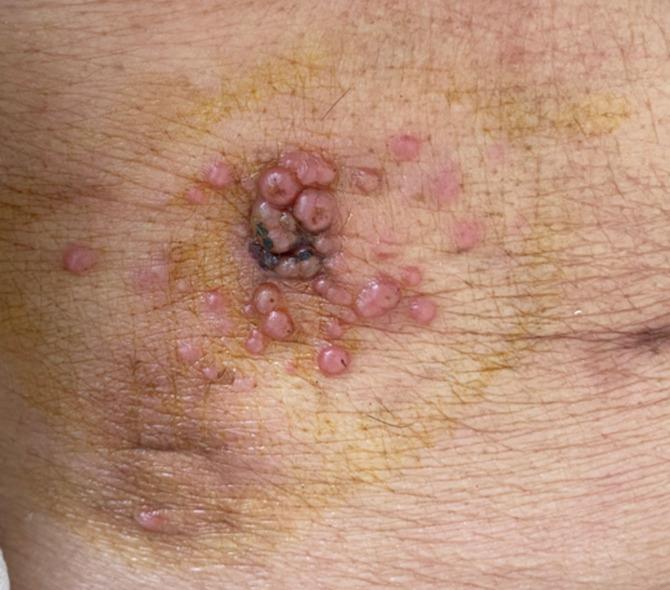
clustering nodules and satellite lesions surrounding the scar

